# Gender and Sex Are Key Determinants in Osteoarthritis Not Only Confounding Variables. A Systematic Review of Clinical Data

**DOI:** 10.3390/jcm10143178

**Published:** 2021-07-19

**Authors:** Matilde Tschon, Deyanira Contartese, Stefania Pagani, Veronica Borsari, Milena Fini

**Affiliations:** Surgical Sciences and Tecnologies, IRCCS Istituto Ortopedico Rizzoli, Via di Barbiano 1/10, 40136 Bologna, Italy; matilde.tschon@ior.it (M.T.); stefania.pagani@ior.it (S.P.); veronica.borsari@ior.it (V.B.); milena.fini@ior.it (M.F.)

**Keywords:** osteoarthritis, sex, gender, men, women, patients, clinical studies

## Abstract

Many risk factors for osteoarthritis (OA) have been noted, while gender/sex differences have been understated. The work aimed to systematically review literature investigating as primary aim the relationship between gender/sex related discriminants and OA. The search was performed in PubMed, Science Direct and Web of Knowledge in the last 10 years. Inclusion criteria were limited to clinical studies of patients affected by OA in any joints, analyzing as primary aim gender/sex differences. Exclusion criteria were review articles, in vitro, in vivo and ex vivo studies, case series studies and papers in which gender/sex differences were adjusted as confounding variable. Of the 120 records screened, 42 studies were included. Different clinical outcomes were analyzed: morphometric differences, followed by kinematics, pain, functional outcomes after arthroplasty and health care needs of patients. Women appear to use more health care, have higher OA prevalence, clinical pain and inflammation, decreased cartilage volume, physical difficulty, and smaller joint parameters and dimensions, as compared to men. No in-depth studies or mechanistic studies analyzing biomarker differential expressions, molecular pathways and omic profiles were found that might drive preclinical and clinical research towards sex-/gender-oriented protocols.

## 1. Introduction

Osteoarthritis (OA) is a widespread musculoskeletal disease, affecting an estimated 300 million people worldwide and >40 million people across Europe. OA is a leading cause of disability, particularly among older adults: an estimated 10% to 15% of all adults aged over 60 suffer from some degree of OA [1,2]. With a constantly increasing elderly population, the treatment of OA has become a major issue to face, with very high costs for healthcare. In fact, no solutions are currently able to effectively arrest or at least slow down the OA degeneration process and treatments options are only capable of symptom alleviation. The onset of OA symptoms is often insidious, showing a remarkable asymmetry of signs (pain, weakness, altered function, stiffness, swelling, instability/buckling, joint enlargement, altered gait, limitation of motion, etc.). Efforts to identify mechanisms responsible for the development, evolution and treatment of OA are still underway today, through the adoption of advanced in vitro models, in vivo investigations and clinical trials [3,4,5,6,7]. In recent years, research has focused on the link between OA and frailty, defined as a state of increased vulnerability to stressors as a result of decreased physiological reserve affecting multiple organs including the musculoskeletal system. The prevalence of frailty increases with age, and it is more frequent in females than in males. Numerous indices are currently in use for the assessment of frailty based on a variety of settings and diseases, related to the “Frailty Phenotype” or the “Deficit Accumulation Index or Frailty Index” models [8]. On one hand, clinical trials have demonstrated that the presence of OA is significantly associated with a higher risk of being frail [9,10,11,12,13,14]; on the other hand, the role of frailty in the development of degenerative joint diseases has been hypothesized due to the increase in pro-inflammatory mediators and the presence of sarcopenia associated with a higher fall risk [15]. While risk factors such as age, obesity, injury and genetic profiles have been identified, the role of gender in OA has been understated [16,17], even if for many years, sex/gender differences have been noted in the prevalence, incidence and severity of OA [18]. Sex is biologically and genetically determined and is binary; gender is a multifaceted expression, that is not binary and encloses roles, behaviors, activities, opportunities that are modeled by the society on a person [19,20]. Although, sex and gender are two different concepts, in medicine they are often linked and in the present study they were used as synonyms.

In 2016, the World Health Organization (WHO) Global Health Estimates estimated that the death/disability rate for musculoskeletal disorders is about 65% in women and 30% in men (www.who.int/healthinfo/global_burden_disease/en/, accessed on 30 March 2021). Studies in literature suggest that women had a higher prevalence of OA, mostly after 50 years of age (increasing dramatically around the time of menopause), and experienced more debilitating pain than men [21,22]. Some authors have investigated the role of estrogen and other hormones as possible explanatory factors, but their results were conflicting [22,23]. The cause could be multifactorial and could include not only hormonal issues, but also genetic factors, anatomic differences and previous injuries [24]. The reasons for such differences are not yet completely clear, but the understanding of how and why OA differently occurs in men and women is crucial for developing effective personalized strategies and refining/improving diagnostic techniques.

Starting from preclinical studies, several authors, and more recently a systematic review, highlighted some important differences in OA between male and female subjects [25,26,27]. For in vitro studies, sex differences emerged at the molecular level and in the gene expression of inflammatory cytokines and hormone receptors. Compared to males, females showed high levels of macrophage stimulators, pro-inflammatory mediators, including inflammatory interleukins, and a higher expression of estrogen receptors. In contrast, elevated catabolic enzymes degrading the extracellular matrix and, at the same time, better compensatory anabolic pathways with an increase in growth factors and testosterone levels were observed in males [28]. This seems to partially reflect the condition observed in the clinic, with greater disability and higher inflammatory status for women compared to men. Regarding in vivo studies, sex differences were found mainly in the histopathological aspects, concerning the OA development and severity, in terms of bone architecture, osteophyte formation, synovial inflammation, cartilage degeneration, deterioration of subchondral bone and pain development [25]. However, in these studies, the lack of analysis of the molecular mechanisms linked to sex and the heterogeneity of the used animal models, produced different and conflicting results which did not allow clear and definitive conclusions to be drawn. The diversity in these studies underlines the need for a synthesis of evidence to clarify if sex and gender factors are relevant in delineating OA. A better understanding of whole spectrum of these differences might contribute to improving the delineation of OA characteristics, the quality of diagnosis, prognosis and care for OA patients.

Therefore, the purpose of the present review was to systematically analyze the current state of knowledge in clinical studies investigating the relationship between gender related discriminants and OA to examine what characteristics are influenced.

## 2. Materials and Methods

### 2.1. Search Question

The PICOS model was used to design this study: (1) studies that considered men and women patients with OA (population); (2) studies where the primary aims were to evaluate sex/gender differences in OA (interventions); (3) studies that presented a control population (women patients vs. men patients) (comparisons); (4) studies that reported morphometric/kinematic/functional/pain/or any other outcomes in OA (outcomes); and (5) clinical studies (study design). Studies from 2010 to 2020 were included in this review if they met the PICOS criteria.

### 2.2. Search Methods

We performed a systematic search on 12 January 2021, carried out according to the Preferred Reporting Items for Systematic Reviews and Meta-Analyses (PRISMA) statement [29]. Three databases were consulted: PubMed (www.pubmed.com), Science Direct (www.sciencedirect.com) and Web of Knowledge (www.webofknowledge.com). The following combination of keywords was used: “osteoarthritis AND (“sex difference” OR “gender difference”)”.

### 2.3. Eligibility Criteria

We included papers following inclusion criteria: clinical reports about sex/gender differences in osteoarthritis conditions, full text, written in English language, published in the last 10 years; moreover, the primary stated item should be the investigation of sex or gender differences in OA population. Exclusion criteria were articles written in other languages, not involving humans, reviews, in vitro, in vivo and ex vivo studies, case series studies, reports in which OA was absent or articles in which gender differences were not taken into consideration as primary aim, as well as abstracts or conference proceedings.

### 2.4. Selection of Studies

Two reviewers (M.T. and D.C.) independently screened the titles and abstracts from the search results to exclude irrelevant articles. After reading the full text of potentially relevant papers, the studies that met the eligibility criteria were included. Disagreements were resolved by discussion. Where resolution was not possible, a third reviewer (S.P.) was consulted. Thereafter, the reference lists of the included papers and relevant reviews on the topic were screened to obtain further studies. Finally, duplicates were removed by submitting papers to a public reference manager (Mendeley 1.14, www.mendeley.com accessed on 22 January 2021).

### 2.5. Data Extraction and Synthesis

The general characteristics of included papers were extracted by two reviewers (D.C. and V.B.), including details of the study (first author’s name and year of publication), number of men and women patients, study design and inclusion criteria, type of surgery, sex prevalence, patient’s mean age, OA diagnosis and site, aim of the study, outcomes, determinants, and main results. Data were extracted into a structured data collection form by one reviewer (D.C.) and were checked for accuracy and completeness by a second one (V.B.). Disagreements were resolved by discussion. Where resolution was not possible, a third reviewer was consulted (M.T.).

### 2.6. Assessment of Quality

The methodological quality of the included papers was assessed by two independent raters (D.C. and S.P.) using the NIH quality assessment tool for observational cohort and cross-sectional studies (https://www.nhlbi.nih.gov/health-topics/study-quality-assessment-tools accessed on 22 February 2021). The tool included 15 items which assessed the possible sources of bias. For each item, we categorized “Yes” if the criterion was explicitly met, “No” if the assessed criterion was not met or “N.A.” if the assessed criterion was unclear, not reported or not applicable. Disagreements were resolved by discussion. Where resolution was not possible, a third rater was consulted (M.T.). A percentage score was used to present the quality assessment results.

## 3. Results

**Figure 1:**
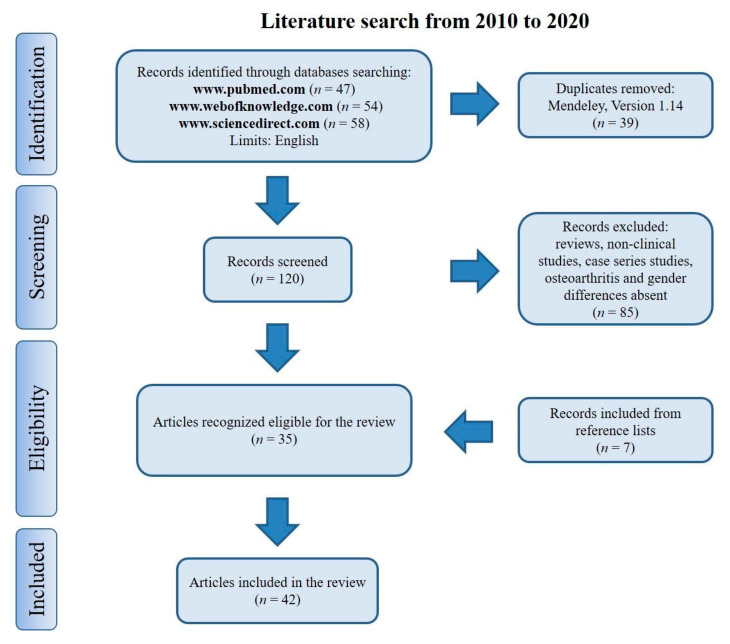
Search strategy according to the Preferred Reporting Items for Systematic Reviews and Meta-Analyses (PRISMA) statement.

The literature search for eligible studies was performed using the previously mentioned keywords: 47 articles were retrieved using www.pubmed.com, 58 articles using www.sciencedirect.com and 54 articles using www.webofknowledge.com. Subsequently, the resulting references were submitted to a public reference manager (Mendeley 1.14, www.mendeley.com to eliminate duplicate articles (*n* = 39). The remaining papers (*n* = 120) were screened for matching the inclusion criteria. Reviews and non-inherent papers including preclinical studies, case series, papers without OA or sex/gender differences (*n* = 85) were excluded. After screening, a total number of 35 articles were recognized as eligible for the review and by examining the reference lists of these studies, 7 other papers were included. A total of 42 studies were included in the review (Figure 1).

The general characteristics of the included studies analyzed based on the PICOS model were extracted in Table 1.

**Figure 2:**
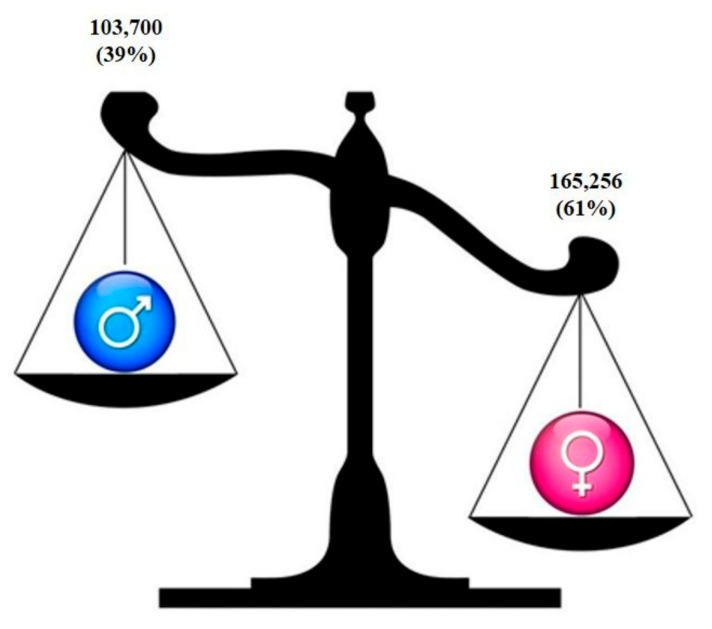
Prevalence of men and women analyzed in the included studies.

Most studies were observational cohort and cross-sectional designed studies; they globally analyzed 268,956 patients, of which 103,700 were men (39%) and 165,256 women patients (61%) (Figure 2).

Sample sizes varied from 30 to 244,059 patients with mild to severe OA diagnosis and a mean age 61.99 (±1.54) years. The most studied joint was knee (33 out of 42 studies, 79%) followed by hip (8 out of 42 studies, 19%) and spine/hand/shoulder (3 out of 42 studies, 7%). OA was diagnosed mainly on X-rays by scoring joints with the Kellgren–Lawrence grading system (22 out of 42 studies) or according to the American College of Rheumatology criteria for the classification and reporting of OA (7 out of 42 studies). Other studies did not report the criteria for OA diagnosis mainly because studies are part of larger RCT and eligibility criteria were published elsewhere or because patients were undergoing arthroplasty and had the more severe OA scenario.

**Figure 3:**
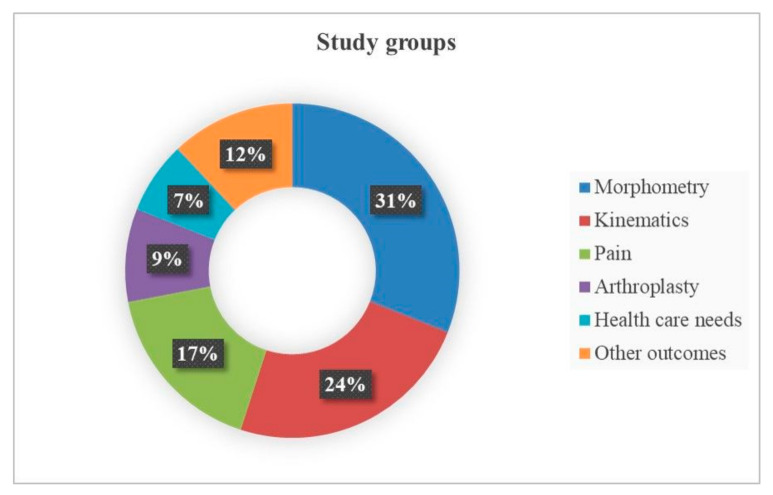
Diagram of percentages of included studies grouped by clinical endpoints investigated.

Since a great heterogeneity in the studies’ aim and clinical endpoints emerged, the included studies were subsequently grouped into six categories based on the type of outcome that was assessed: studies investigating morphometry (*n* = 13), kinematics (*n* = 10), pain experience (*n* = 7), arthroplasty (*n* = 4), health care needs (*n* = 3), and other outcomes (*n* = 5) (Figure 3).

### 3.1. Data Extraction

#### 3.1.1. OA and Morphometry

Most of the studies (31%) evaluated gender differences of OA patients in relation to hip, hand, spinal, femoral and tibia morphometry, and to changes in bone microarchitecture and remodeling. In addition to age and BMI, other major determinants in these studies are race and ethnicity: the study populations included Korean, Chinese, Japanese, Singaporean, African American (AA) and White (WH) patients and used radiographs, computed tomography (CT) scans or magnetic resonance imaging (MRI). The adopted scores were: ATLAS score for disc space narrowing and osteophytosis, Kellgren–Lawrence (K-L) and joint space narrowing (JSN) grades. 

Overall, these studies analyzed *n* = 8949 patients, 5975 (67%) women and 2974 (33%) men. Results showed that, at knee level, the medial joint space loss was higher in AA men than in WH and AA women. Differently, compared to WH women, WH men had lower risk of K-L grade worsening [30]. In evaluating the sensitivity of some radiographic measurements (femorotibial angle, FTA; hip–knee–ankle angle, HKA; goniometry) to medial and lateral JSN, the offset between FTA measures and HKA was larger in women than men [31]. As for femur morphometry, women had narrower and smaller femurs [32,33,34], reduced femoral offset values, but a greater valgus neck-shaft angle compared to men [35]. In addition, no difference in the higher varus malalignment and in the lower femoral neck anteversion, and no alteration in the femoral anterior bowing, was found between men and women [36]. As for tibia morphometry, mediolateral width (ML), middle anteroposterior length (AP), ML/AP aspect ratio, medial anteroposterior (MAP) and lateral anteroposterior (LAP) were higher in men compared to women. The MAP was larger than LAP, and there was a positive correlation between ML and AP dimension and a negative correlation between the ML/AP aspect ratio and AP dimension both in men and women [37]. In hip morphometry, men tended to have greater acetabular depth and width, AP alpha and Gosvig angles, minimum joint space width, extrusion and acetabular indices, and modified triangular index height, as well as more frequent triangular index sign. Women tended to have greater, lateral center edge angle and more frequent protrusio acetabuli and coxa profunda [38]. In addition, no sex differences were found for both microarchitecture and bone remodeling in subchondral trabecular bone (STB). However, differences were found in deeper trabecular bone (DTB), with thinner trabecular thickness (Tb.Th), higher trabecular number (Tb.N), higher specific osteoid surface (OS/BV) and specific eroded surface (ES/BV) in men than women. In both STB and DTB, no correlation between microarchitecture and age was found in both sexes, unlike bone remodeling. The latter in fact, in STB increased with age in men but not in women, while in DTB increased with age in women but not in men. No age or gender preference was found in subchondral bone cyst frequency and volume fraction [39]. Finally, women, compared to men, had much higher OA prevalence [40,41]. At the spine, ATLAS score did not show differences between men and women, but only differences in the anatomical involvement: women had mostly thoracic involvement and men mostly lumbar involvement [42].

#### 3.1.2. OA and Kinematics

Several studies (24%) evaluated gender differences in kinetics and kinematics of patients with knee or hip OA. Here, major determinants are age, BMI and mostly the walking speed; indeed, the retrieved studies analyzed patients suffering with varying grades of OA severity, from early to severe, making comparisons difficult. Performed analyses included MRI, three-dimensional gait analysis, and physical function tests (timed Up and Go (TUG), stair climbing test, and 6-min walk test (6 MWT)). Overall, these studies analyzed *n* = 1290 patients, 720 (56%) women and 570 (44%) men. Apart from a single study where no differences were found in any gait kinematic variable between OA and healthy patients in either men or women [43], all other studies showed gender differences. Men walked with a greater forward trunk lean and had greater peak hip external rotation moment than women [44]. However, women had a greater external hip adduction moment throughout stance than men [44,45]. Compared with patients with pain in the anterior knee compartment, those with pain in the medial compartment exhibited a slower walking speed, shorter step length, and lower single-limb-support phase. These differences are witnessed mainly among women, whereas men differed only in single-limb-support [46]. Men had a higher absolute peak knee adduction moment (KAM), KAM impulse, peak vertical ground reaction force (GRF) compared to women [47]. Therefore, in patients with OA, gender influenced the variability of cadence, knee and hip motion, pelvis rotation, and variability of step length [48]. Finally, regarding muscle activity, women had greater biceps femoris and gastrocnemius activity during respective lateral and anterior–medial loading directions compared to men [49], greater quadriceps and hamstrings strength, but less activity on the TUG test, stair climbing test, 6 MWT [50], rate of torque development of the knee extensors [51], muscle volume, velocity, power and peak twitch tension [52].

#### 3.1.3. OA and Pain 

Some studies (17%) evaluated sex/gender differences in pain severity or intensity in patients with knee and hip OA. Many factors that can contribute to the pain status were considered as determinants such as family history of OA, education, marital and occupational status, presence of comorbidities, and drug assumption. The outcomes were measured using MRI scans for determining cartilage volume and signs of synovitis, visual analog scale (VAS), quantitative sensory tests including psychosocial measures, function tasks and physical activity, but also by measuring endogenous sex hormones (estradiol, progesterone, testosterone) and inflammatory markers (Interleukin (IL)-6, IL-8, IL-10, IL-1β, tumor necrosis factor (TNF)-α, metalloproteinase (MMP)-10 and C-reactive protein (CRP)). Overall, these studies analyzed *n* = 3583 patients, 2100 (59%) women and 1483 (41%) men. Results showed that women reported a worst clinical pain scenario, with higher VAS, higher painful knee count, greater serum CRP concentration, and more impaired function compared to men. In particular, in women but not in men, low serum levels of endogenous estradiol, progesterone and testosterone were associated with increased pain and knee effusion-synovitis volume and decreased cartilage volume [53]. By analyzing synovial fluids, MMP-10 and other chemokines (IL-8, CCL-4, monocyte chemoattractant protein (MCP)-2) were higher in men, whereas inflammatory cytokines (IL-6, IL-10, IL-1β, TNF-α) were higher in women, confirming they experienced more pain than men [54,55]. However, psychosocial measures (depression, anxiety, social support) and physical activity were similar between genders [54,56,57,58]. Finally, one study [59] used a “single-blind placebo lead-in” design to evaluate the placebo response to pain, depressive and anxiety symptoms, and performance-based tests, in patients with knee OA. Results showed that men reported greater depressive symptoms than women, while concerning performance, women showed greater walking resistance than men.

#### 3.1.4. OA and Health Care Needs

Few studies (7%) evaluated sex/gender differences in the utilization of health care resources, of OA patients scheduled for total hip or knee arthroplasty, by applying questionnaires. Several gender aspects such as marital and occupational status, presence of insurances and region of origin were considered issues relevant related to the health care needs. Overall, these studies analyzed *n* = 249,839 patients, 152,969 (61%) women and 96,870 (39%) men. Results showed that compared to men, women (1) ask a greater number of questions, particularly on their condition and surgical procedure and related risks and benefits [60]; (2) are more likely to be referred to specialist care, consult orthopedic surgeon, or to be on waiting list for total hip replacement [61]; and (3) are more likely to receive a narcotic and non-narcotic analgesics, corticosteroid and hyaluronic acid injection, MRI imaging, physical and occupational therapy [62].

#### 3.1.5. OA and Arthroplasty

Some studies (9%) evaluated sex/gender and age differences in patients-reported outcomes and improvements in OA patients, mostly Japanese and Korean, undergoing total arthroplasty of knee, shoulder, or hip. Major determinants were age, BMI, K-L grading and even smoking habits that negatively affect the bone stock quality thus representing a relevant issue in the arthroplasty success rate [63,64]. Totally, *n* = 2866 patients were evaluated, 2011 (70%) women and 857 (30%) men. Evaluations were made mainly by radiographs, several questionnaires, and scores (2011 revised Knee Society Score (KSS2011), Knee Society Score, Knee injury and Osteoarthritis Outcome Score (KOOS), Oxford Knee Score, VAS, 12-Item Short Form Health Survey, Short Form 36, American Shoulder and Elbow Surgeons scores, Western Ontario and McMaster Universities Arthritis Index (WOMAC), Harris hip score). Results showed that both KSS2011 and KOOS scores decreased with age and particularly in women over 50 [65]. Despite an improvement in women [66,67], men however achieved higher scores concerning functional and physical component of knee and hip measured with the other scores, while there was no difference between men and women in shoulder pain and mental outcomes [68]. OA negatively affects the quality of life and physical function of both genders, even if women are more adversely affected than men.

#### 3.1.6. OA and Other Outcomes

Some studies (12%) evaluated sex/gender differences in prevalence of knee and spinal OA in patients, mostly Korean and Japanese, and the association between OA and other related disorders as neuroinflammation or single and multiple falls. These studies analyzed *n* = 2429 patients, 1481 (61%) women and 948 (39%) men. Assessments were made mainly by radiographs or CT scan, functional scores such as the WOMAC for physical difficulty and pain severity and performance-based tests (6 MWT and chair stand times). Results showed that women, compared to men, had much higher pain and tended to report more physical difficulty and more impaired function of knee. However, psychosocial measures (depression, anxiety, social support) did not differ between genders [69,70,71]. Compared to men, women more reported at least one or multiple falls. In particular, knee pain, vertebral fracture and a longer 6 MWT were risk factors for multiple falls in women, while a longer chair stand time was a risk factor for multiple falls in men [72]. Gender differences were also found in cytokines involved in blood borne, neuroimmune joint-to-central nervous system (CNS) signaling. There were positive correlations for MCP-1 in cerebrospinal fluid, serum, and synovial fluid, in women, but not in men. Symptom severity correlated with IL-6 and IL-8 levels in synovial fluid but was inversely associated with IL-6 and IL-8 levels in cerebrospinal fluid, indicating that neuroinflammation in OA may be an adaptive, possibly neuroprotective mechanism promoting symptom reduction [73].

### 3.2. Quality Assessment

**Figure 4:**
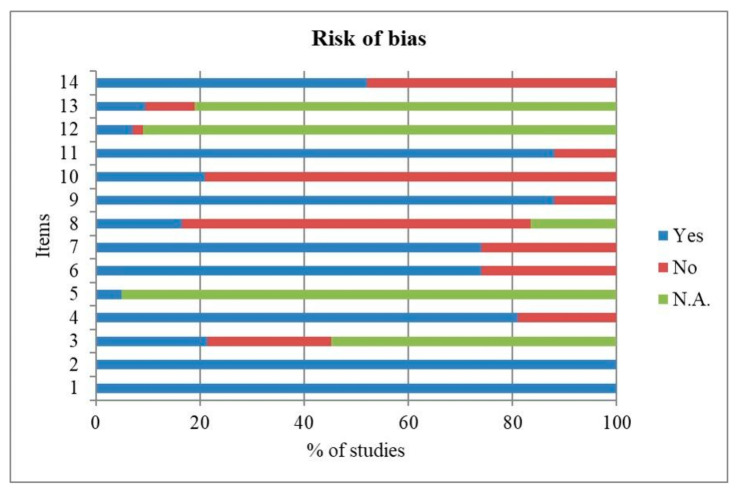
Frequencies (%) of risk of bias assessment according to the National Institutes of Health (NIH) quality assessment tool for observational cohort and cross-sectional studies (https://www.nhlbi.nih.gov/health-topics/study-quality-assessment-tools).

Quality and risk of bias assessments for clinical studies are shown in Figure 4.

The results of the National Institutes of Health (NIH) quality assessment tool for observational cohort and cross-sectional studies (https://www.nhlbi.nih.gov/health-topics/study-quality-assessment-tools) showed a high risk of bias for items 8 on “different levels of the exposure as related to the outcome” and 10 on “exposure(s) assessed more than once over time”, since the criteria were not met with 67% and 79% of frequencies, respectively.

A low risk of bias was estimated for items 1 on “clarity of the research question or objective”, 2 on “study population clearly specified and defined”, 4 on “clarity of inclusion and exclusion criteria ”, 6 on “exposure(s) of interest measured prior to the outcome(s)”, 7 on “sufficient timeframe”, 9 on “reliability of exposure measures”, 11 on “reliability of outcome measures” and 14 on “key potential confounding variables measured and adjusted statistically” because the criteria were explicitly met, with 100%, 100%, 81%, 74%, 74%, 88%, 88% and 52% of frequencies, respectively. The remaining items 3 on “participation rate at least 50%”, 5 on “sample size justification and power description provided”, 12 on “blinding of outcome assessors”, and 13 on “loss to follow-up less than 20%”, presented an unclear risk of bias, since the criteria for judging were unclear, missing, not reported or not applicable.

## 4. Discussion

From an epidemiologic perspective, it would be relevant to know how and to which extent gender/sex related differences influence health and, in particular, might contribute to the development and treatment of OA. This aspect becomes even more prominent from a diagnostic, prognostic and therapeutic perspective, because the recognition of sex-based differences would lead to the development of ad hoc diagnostic approaches and offer sex-personalized therapeutic protocols for OA patients, even more in a so heterogeneous pathology such as OA. So far, the aim of the present review was to systematically analyze clinical studies particularly focused on evaluating gender and sex differences in OA.

From this screening of the literature, it becomes clear that there is significant heterogeneity across the studies in the outcomes assessed to investigate these differences. Main outcomes that have been investigated in terms of gender and sex differences are morphometric accounting for 31%, studies on gait and kinematics for 24%, on pain for 17%, on functional and performance outcomes after arthroplasty for 9%, on health systems demand for 7% and other mixed outcomes for 12% of studies. In terms of clinical relevance, morphometric studies are significant not for OA diagnosis but in understanding the geometry and anatomy, for guiding surgeons during arthroplasty for the correct positioning of the implants and, therefore, are relevant for determining the longevity of implant fixation. These studies revealed population differences with a lower risk of OA worsening in Caucasian men than women. Femoral and tibial differences were highlighted with smaller dimensions and curvatures in women than men. Subchondral bone microarchitecture was not affected by sex, whereas the deeper trabecular bone had thinner thickness and a higher number of trabeculae in men than in women. Most studies performed morphometric measurements in the operating room at time of arthroplasty, meaning that patients were affected by the end stage of OA, thus reflecting the worse OA scenario.

In kinematics studies, sex influenced the variability of cadence, knee motion, hip motion, pelvis rotation, and variability of step length also due to the gender differences present in the muscle pattern and performance in term of increased muscle volume, velocity and power in men than women. These findings about sex-specific patterns of movements might be taken into consideration for surgical approaches, treatments or rehabilitation protocols for OA patients after arthroplasty procedures or in relation to falls and injury mechanisms.

In studies investigating pain experience differences between females and males, the complexity of evaluating pain which encloses many clinical signs and subjective outcomes must be considered. Undoubtedly, women reported worst pain scenario, with increased VAS, serum CRP, inflammatory cytokines and more impaired function compared to men. Accordingly, to a worst pain, women are more willing to benefit from health systems and caregivers, by consulting specialists, asking for procedures, risks and benefits and drugs. The perception and measurements of pain have been debated and investigated: some studies concluded that women had lower sensation thresholds and pain thresholds than men [74,75]. Taken together, the results indicate that pain appears to be understudied; reasons could be related to its complex nature. In fact, multiple biological, genetic, ethnic, emotional, environmental and psychosocial factors are reported to contribute to pain and pain sensitivity in OA patients [76,77,78,79]. Chronic pain due to OA can be nociceptive caused by peripheral sensitization mostly due to structural and anatomical damages or neuropathic when an abnormal pain sensitization mediated usually by the central nervous system occurs [80]. Thus, pain is difficult to evaluate objectively because of its multifactorial nature and of the variability in the patient’s threshold. Pain threshold measurements are commonly performed by pressure, electrical or thermal responsiveness or by delivering self-reported questionnaires [80,81] of which the most frequently used are the WOMAC, VAS or Likert scales. Diagnostic imaging is recommended to confirm the diagnosis of OA, even though it has poor correlation with clinical pain experienced by the patient [82]. The link and involvement of sexual hormones on pain has been clearly demonstrated; in fact, estrogens mitigate pain in a dose dependent manner and testosterone reduces the sensitivity to chronic pain [83,84]. Moreover, hormones and their receptors have protective effects on articular cartilage biochemistry, and this might be the cause of greater progression of OA in women after menopause [66,85]. These observations, coupled with lower cartilage thickness in women than in men [86], might explain why women lose articular cartilage at three to four times the annual rate of men [87] and why women present greater OA severity. So far, due to the great heterogeneity of records on pain, evidence-based conclusions cannot be drawn.

After arthroplasty, men achieved better and faster functional and physical recoveries, independently of the substituted joint. Total knee substitutions had poorer final functional outcomes particularly in Caucasian women, probably owing to a more advanced stage of disease, greater age and more muscle atrophy at time of surgery than men [66]. These results are concordant with the work of O’ Connor et al. who tried to identify potential sex differences with respect to implant survival, function and pain after arthroplasty. They concluded that women have a more favorable prognosis but with lower functional scores and worse pain before and after knee arthroplasty [87].

In relationship to the other outcomes evaluated in the included studies, it appeared that psychosocial measures, such as depression, anxiety, and social support, did not differ between genders. Instead, women had a higher risk for multiple falls due to pain and vertebral fractures that might be related also to a depleted estrogen status. Moreover, obesity and aging were found to be associated with the risks of knee OA, but female sex was the strongest risk factor, followed in order by obesity and aging [40]. In addition to sociodemographic factors, genetic and environmental risk factors, such as age, BMI, race, disease familiarity, physical activity, as well as local joint related risk factors, such as site, bone and muscle anatomy, are major determinants in affecting OA that have to be undertaken [18,88].

Individuals with OA have a high incidence of comorbidities triggering a profound impact on OA treatment and management, patients’ quality of life, healthcare provision and costs [89]. Not only are individuals with OA 1.2 times more likely to have any comorbidity than non-OA ones and 2.5 times more likely to have ≥3 comorbidities [90] but a greater comorbidity burden is reported to worsen pain and physical function in people with knee and/or hip osteoarthritis in the meta-analysis study by Calders and Van Ginckel [91]. The presence of comorbidities in patients affected by OA was evaluated in 6 out of the 47 studies included in this review dealing with health care needs [61,62], arthroplasty [68] and pain [55,56,57]. The presence of comorbidities was analyzed by the Charlson Comorbidity Index [56,62,68], patient reporting on chest tightness, wheeze, breathlessness, chest pain or palpitations [61], or the number of concurrent medical conditions as indicated by no/yes responses to the American Academy of Orthopedic Surgeon’s Comorbidity scale [55,57]. No significant differences were found between men and women in these studies, even though Juni et al. found that women tended to be less likely to report severe comorbidity than men, only 6% of women vs. 8% of men [61].

In the included studies a sex/gender misbalance was noted, since 39% of analyzed patients were male and 61% were female; this misbalance remained also when papers were sub-grouped in the different observed outcomes. Fortunately, this aspect is in contrast with data of past years in which women remain underrepresented in biomedical research and sex bias exists within enrolment in clinical trials, with many more men recruited than women [71,92]. Even preclinical research is conducted preferentially in male animals and in vitro tests and in vivo investigations that perform assays on cells and animals of both sexes are rare [25].

**Figure 5:**
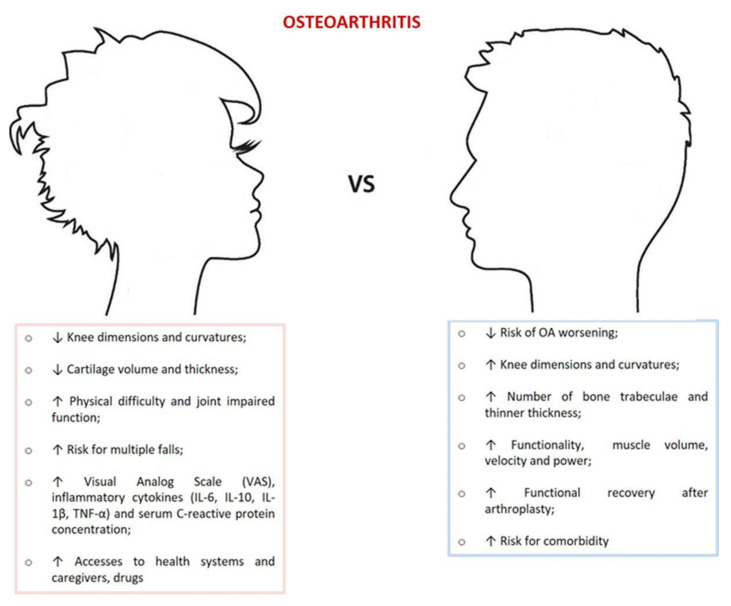
Major findings between men and women in OA.

Limits of the present review are related to the difficulty in retrieving clinical works in which the primary aim and primary outcome measure were clearly stated to investigate sex differences. In fact, gender differences might be undetected because of typical approaches adopted in most clinical studies that consider gender and sex as confounding variables and report effects from sex-adjusted analyses. Several other flaws could be identified: among them, the inclusion of many different designed studies and including different outcomes. Heterogeneity among studies remained an important factor limiting the interpretation of our results. Indeed, we limited our search to clinical studies even because in a previous systematic review [25] we analyzed sex differences existing in the in vitro and in vivo scenario. However, we performed a comprehensive search and summarized the gender and sex determinants that have been investigated by the different studies in OA patients, that in the authors’ opinion well represent the multifaceted aspect of OA. Although our findings are predominantly based on results from cross-sectional and cohort studies, we identified main characteristics, as shown in Figure 5. Further studies analyzing patients more homogeneous for risk factors even with meta-analysis are required to measure how and to which extent gender and sex differences affect the prevalence, incidence and severity of OA.

The inclusion in this study only of papers reporting sex differences in the primary outcome measure strengthens the work design focused to determine whether the patient’s sex and gender impacts on OA. The selection, data extraction and risk of bias assessments were conducted independently by two assessors with a consensus check performed by a third assessor; this is a strength of this review. Results were also difficult to interpret because the variation in joint sites of the retrieved studies that might have not revealed sex differences; for example, insufficient data were retrieved for hand, spine and shoulder OA.

Even if the search for “osteoarthritis” and “sex” or “gender” in the www.clinicaltrialg.gov website (accessed on 25 March 2021) gave 99 and 93 study results, respectively, only four clinical trials effectively had, as a primary outcome measure, the analysis of sex differences. There are two completed studies aimed at comparing functional outcome, radiographic results, range of motion, patient satisfaction, and fit of gender-specific versus traditional knee replacement systems design. Another one supposed that acute postoperative pain is different over time between males and females also in terms of responses to multimodal analgesic regimens after arthroplasty. One trial investigated if sex differences exist in the presence of inflammatory mediators or in responses of osteoblasts and chondrocytes to Vitamin D3 and estradiol or in the presence of content and distribution of neural markers in synovial tissue, menisci and cartilage that could respond to the greatest pain experienced by women.

From retrieved papers, there is a lack of studies investigating possible mechanisms of actions, on prognostic and diagnostic biomarkers differential expressions, on genetic determinants, molecular pathways and omic-based analyses; neither trials on sex-targeted drugs were retrieved. Instead, from a laboratory point of view, biological, mechanistic and molecular patterns that could explain gender and sex differences significatively appreciated at clinical level are of outmost importance. Moreover, in the era of big data, omics studies (transcriptomics, epigenomics, proteomics and metabolomics) performed on male and female samples might reveal valuable sex distinctive changes, shedding light on “sexomics and genderomics” [19].

The consolidation of knowledge on these mechanisms and features could help in diagnosing the disease, in stratifying patients and in developing new gender-based approaches, ranging from physical rehabilitation programs to sex-specific drugs for OA.

## 5. Conclusions

Although our findings are predominantly based on results from cross-sectional and cohort studies, we identified gender differences in joint morphometry, kinematics, pain severity, use of healthcare resources, and functional recovery after arthroplasty. Women appear to use more health care, have higher OA prevalence, clinical pain and inflammation, decrease cartilage volume, physical difficulty, smaller joint parameters and dimensions as compared to men.

In the era of precision medicine, more high-quality prospective clinical and preclinical studies that elucidate sex- and gender-based determinants are needed for implementing the diagnosis, development and treatment of male and female OA, toward the definition of gender-oriented protocols.

## Figures and Tables

**Table 1:** Clinical studies.

N. of Patients/Groups	Study Design and Inclusion Criteria	Surgery Type	OA Diagnosis and Site	Sex Prevalence (%)	Age (Mean ± SD)	Aim	Outcomes	Determinants	Main Results	Author (Year)
*Morphometry*										
1882 patients (786 men and 1096 women): (1) WH patients = 1623 (694 men and 929 women); (2) AA patients = 259 (92 men and 167 women)	Cohort study AA and WH individuals with or at risk for symptomatic KOA, K-L grade 0 or 1 and aged 45–79 years	-	X-rays (OARSI and K-L grading system) Knee	42% (men) and 58% (women)	(1) 59.1 (±9.2) years (men) and 60.1 (±9.1) years (women) (2) 57.0 (±9.0) years (men) and 56.8 (±7.9) years (women)	To characterize radiographic worsening in OA by race and sex over and evaluate role of risk factors	K-L and OARSI grade, JSN and fJSW	Age, race, BMI and family history of knee replacement surgery	↑ fJSW in men vs. women, at baseline (group 1 and 2). ↓ risk of radiographic K-L grade and of worsening lateral JSN in men vs. women (group 1). ↑ risk of medial JSN worsening based on OARSI grade progression in men (group 2) vs. women (group 1). ↑ medial fJSW in men, at 4 years (group 2) vs. men (group 1) and women (group 1 and 2)	Vina ER (2018)
2123 patients (885 men and 1238 women): (1) patients with medial JSN = 1063 (484 men and 579 women); (2) patients without JSN = 805 (306 men and 499 women); (3) patients with lateral JSN = 255 (95 men and 160 women)	Cross-sectional study Individuals with or at high risk for developing knee OA	-	X-rays, OARSI and K-L grading system Knee	42% (men) and 58% (women)	(1) 62 (±9) years (men) and 63 (±9) years (women) (2) 60 (±9) years (men) and 60 (±8) years (women) (3) 61 (±10) years (men) and 65 (±9) years (women)	To explore cross-sectional relationships between radiographic measures, quantify sex differences and evaluate sensitivity to medial and lateral JSN	FTA, HKA and goniometry	Age and BMI	↑ Correlation between HKA and FTA in women vs. men (group 3). ↑ offsets for all measurement comparisons except between goniometry and HKA in women vs. men (group 2 and 3)	Moyer R (2015)
352 Asian patients (62 men and 290 women)	Cross-sectional study Asian patients with OA	TKA	Not reported Knee	18% (men) and 82% (women)	-	To investigate sex differences in distal femoral dimensions	AP lateral and medial, ML width and AP/ML ratio	Race	↑ AP medial and lateral, ML width in men vs. women. ↑ AP/ML ratio and narrower femurs in women vs. men	Chin PL (2011)
1025 Korean patients (50 men and 975 women)	Cohort study Korean patients with primary OA	TKA	Not reported Knee	5% (men) and 95% (women)	69.2 (±7.5) years (men) and 68.7 (±6.1) years (women)	To report sex differences in anthropometric features of femurs and their implications in functional outcomes	Condylar and trochlear ML widths, condylar AP height and ML/AP ratio	Age, height, weight and BMI	Femora smaller and narrower in women vs. men. = ML/AP ratio in men and women. ↑ condylar AP height, condylar and trochlear ML widths, condylar underhang in men vs. women. ↓ condylar overhang in men vs. women	Chung BJ (2015)
1168 Korean patients (302 men and 866 women)	Cohort study Korean patients with OA	TKA	Not reported Knee	26% (men) and 74% (women)	69.0 years (men) and 67.4 years (women)	To obtain and compare anthropometric data on knees with dimensions of available TKAs	AP, ML and TEA of femur and tibia	Age, weight and BMI	TKAs do not provide a reasonable fit for knees	Ha CW (2012)
664 patients (190 men and 474 women): (1) patients with lateral OA = 160; (2) patients with medial OA = 168; (3) controls = 336	Case-control study Individuals with or at elevated risk of knee OA, OARSI ≥2, K-L ≥1 and aged 50–79 years	-	X-rays (OARSI and K-L grading system) Knee	29% (men) and 71% (women)	63.3 (±8.2) years	To explore sex differences in hip and pelvic geometry, in the presence of compartment-specific knee OA	Hip/pelvic geometry and knee alignment (ABD angle, NSA, FNL, HHC, BWLA, ABD, FH-FH length and HKA)	Age, height and BMI	↓ FO in women vs. men. ↑ NSA and HKA in women vs. men	Boissonneault A (2014)
214 patients (54 men and 160 women): (1) patients with OA = 169 (33 men and 136 women); (2) healthy patients = 45 (21 men and 24 women)	Case-control study Subjects with radiographic knee OA, K-L grades 3–4 and primary TKA for varus OA	-	X-rays (K-L grading system) Knee	25% (men) and 75% (women)	(1) 74.9 (±5.2) years (2) 65 (±4.9) years	To assess sex differences in femoral alignment and deformity in patients with varus knee OA	Femoral lateral and anterior bowing, neck anteversion, HKA and torsion angle	K-L grade and recruiting hospital	↑ Femoral lateral bowing, neck anteversion, change of the angle of DL relative to P1/3 in women vs. men (group 1)	Mochizuki T (2017)
130 Chinese patients (65 men and 65 women)	Cohort study Chinese patients with OA	-	Not reported Knee	50% (men) and 50% (women)	61.4 (±8.3) years (men) and 61.6 (±7.7) years (women)	To compare morphologic and geometric differences of proximal tibia between men and women	Tibial ML, AP, LAP, MAP and ML/AP ratio	Age and height	↑ ML, AP, LAP, MAP, ML/AP ratio in men vs. women. More oval shaped tibial prosthesis in men vs. women	Yang B (2013)
71 patients (17 men and 54 women): (1) AA patients = 20; (2) WH patients = 51	Case-control study AA and WH idividuals with OA and K-L grade < 3	-	X-rays (K-L grading system) Hip	24% (men) and 76% (women)	63 (±8) years	To describe effect of alterations in hip morphology with respect to worsening OA	AD, AW, AD/AW, AI, extrusion and triangular index, LCEA, protrusio acetabula, coxa profunda, APα angle, Gosvig ratio, MTHI, PFA, FSA, femoral head and neck, interacetabular edge, distance between femoral heads and teardrops, and SI joint to pubic symphysis	Age, race, side (left/right hip), BMI, height, weight and history of hip injury	=APα angle in men and women. ↑ APα angle >60, PFA, protrusio, profunda and triangular index in women vs. men. ↓ minimum JSW, Gosvig ratio and AI in women vs. men	Nelson AE (2016)
110 patients (60 men and 50 women): (1) patients with moderate OA = 22 (12 men and 10 women); (2) patients with severe OA = 88 (48 men and 40 women)	Cohort study Patients with for late-stage primary, moderate or severe OA	-	X-rays (K-L grading system) Hip	55% (men) and 45% (women)	64.63 (±11.72) years (men) and 69.16 (±12.33) years (women)	To investigate age- and sex-related changes of microarchitecture and bone remodeling in OA subchondral bone	SBC, CV/TV, BV/TV, Tb.Th, Tb.Sp, Tb.N, SMI, DA, Conn.D, BMD, in STB, DTB, O.Th, OV/BV, OS/BS, OS/BV, ES/BS, ES/BV, ES/TV and OS/ES	Age and K-L grade	↑ BV/TV, Tb.Th, Tb.N, Conn.D, BMD, O.Th, OV/BV, OS/BS, OS/BV, ES/BS, ES/BV and ES/TV in men and women (in STB vs. DTB). ↓ Tb.Sp, SMI and DA in men and women (in STB vs. DTB). No gender differences in STB. ↓ Tb.Th in men vs. women (in DTB). ↑ Tb.N, OS/BV, ES/BV in men vs. women (in DTB). No gender and age difference for CV/TV and SBC	Li G (2015)
696 Korean patients (298 men and 398 women): (1) patients with K-L grade 2; (2) patients with K-L grade 3; (3) patients with K-L grade 4, severe symptoms and WOMAC score > 44	Cohort study Korean individuals 65 years or older with knee OA, K-L grade 2, 3 or 4 and WOMAC score >44	TKA	X-rays (K-L grading system) Knee	43% (men) and 57% (women)	71.7 (±5.3) years	To document sex differences in the involvement and prevalence of different stages of OA	WOMAC and K-L score for OA prevalence and involvement (unilateral/bilateral)	Age, height, weight and BMI	↑ Bilateral involvement and prevalence for all stages of OA in women vs. men	Cho HJ (2011)
97 patients (32 men and 65 women): (1) patients with early OA = 38 (7 men and 31 women); (2) asymptomatic patients = 59 (25 men and 34 women)	Cross-sectional study Patients with early OA	-	Not reported Hand	33% (men) and 67% (women)	(1) 56.3 (±6) years (men) and 53.9 (±6.8) years (women) (2) 36.8 (±13.6) years (men) and 42.3 (±16.4) years (women)	To determine correlation of the first CMC joint with sex and early onset of OA	Joint shape and size	Age	No correlation of joint congruence with OA and sex for joint size	Conconi M (2014)
417 patients (173 men and 244 women)	Cohort study Individuals with spinal OA	-	X-rays Spine	41% (men) and 59% (women)	50 (21–101) years (men) and 51 (20–82) years (women)	To characterize differences in prevalence and distribution of spinal OA between men and women	ATLAS score, DSN and OST	Age and BMI	=Score in men and women. ↑ DSN in men vs. women. ↑ OST in men (in thoracolumbar joint and lumbar regions) and women (in mid thoracic region)	Duncan AE (2012)
*Kinematics*										
100 patients (45 men and 55 women)	Cross-sectional study Subjects with mild-to-moderate knee OA, K-L grade < 3, VAS score >20 mm and aged 33–72 years	TKA	ACR clinical criteria Knee	45% (men) and 55% (women)	55.18 (±7.54) years (men) and 55.33 (±7.26) years (women)	To examine sex differences in gait kinematics at ankle, knee, hip joints, foot and pelvis segments between patients with knee OA	Kinematic joint angles	Age, height, weight, BMI and walking speed	↑ Knee abduction at touchdown and during swing, maximum peak hip adduction angle during stance in women vs. men	Phinyomark A (2016)
66 patients with mild-to-moderate OA (28 men and 38 women)	Cross-sectional study Participants with unilateral mild to moderate hip OA and K-L grade 2 or 3	-	ACR clinical criteria and x-rays (K-L grading system) Hip	42% (men) and 58% (women)	59.4 (±8.7) years (men) and 62.4 (±7.7) years (women)	To investigate association between sex-specific hip kinetics and early-mid stage OA	Hip joint and moments, trunk and pelvic angles	Age, height, weight, BMI, K-L grade and walking speed	↑ External hip adduction moment and angles in women vs. men. Men walk with a greater forward trunk lean vs. women	Allison K (2018)
309 patients (119 men and 190 women): (1) patients with OA = 150 (64 men and 86 women); (2) asymptomatic patients = 159 (55 men and 104 women)	Cross-sectional study Subjects with symptomatic hip OA	-	X-rays (K-L grading system) Hip	39% (men) and 61% (women)	(1) 60.6 (±10.8) years (men) and 63.5 (±9.1) years (women) (2) 55.8 (±8.0) years (men) and 55.6 (±8.6) years (women)	To investigate sex differences in gait associated with OA	Sagittal plane hip range of motion, peak external flexion and extension moments	Age, BMI, K-L grade and walking speed	↑ Sagittal plane hip range of motion and extension moment in women vs. men (group 1 and 2). ↓ flexion and adduction moments in women vs. men (group 1 and 2). ↑ external and internal rotation moments in women vs. men (group 1)	Foucher KC (2017)
240 patients (106 men and 134 women): (1) patients with medial pain = 170 (75 men and 95 women); (2) patients with anterior pain = 70 (31 men and 39 women)	Cross-sectional study Patients with symptomatic bilateral knee OA and medial or anterior knee pain	-	ACR clinical criteria Knee	44% (men) and 56% (women)	(1) 61.5 (±10.7) years (men) and 63.2 (±9.0) years (women) (2) 63.0 (±8.7) years (men) and 62.2 (±8.7) years (women)	To compare gait patterns of patients with OA and anterior or medial joint pain	Walking velocity, step length and SLS	Age, height, weight and BMI	↑ Walking velocity, step length and SLS in men vs. women	Debi R (2012)
35 patients (18 men and 17 women)	Cohort study Patients with severe knee OA	TKA	Not reported Knee	51% (men) and 49% (women)	69.7 (±6.9) years (men) and 70.9 (±8.2) years (women)	To examine sex- and obesity-related differences in knee biomechanics of patients with severe OA	Impulse and absolute peak KAM, peak flexion moment, varus-valgus thrust and angles, and vertical GRF	Age, height, weight, BMI and walking speed	=Peak flexion moment and kinematic variables in men and women. ↑ impulse and absolute peak KAM, vertical GRF in men vs. women	Paterson KL (2017)
110 patients (54 men and 56 women): (1) patients with moderate OA = 45 (24 men and 21 women); (2) patients with severe OA = 45 (22 men and 23 women); (3) healthy patients = 20 (8 men and 12 women)	Cross-sectional study Patients with unilateral moderate or severe knee OA, K-L grade 3 or 4 and aged over 65 years	-	X-rays (K-L grading system) Knee	49% (men) and 51% (women)	(1) 70.1 (±3.6) years (men) and 68.4 (±2.5) years (women) (2) 67.6 (±3.3) years (men) and 69.3 (±4.2) years (women) (3) 71.9 (±2.8) years (men) and 69.4 (±3.4) years (women)	To clarify association between sex and gait parameters in severe OA	KOOS score, cadence, step length, knee and hip angle, and pelvis rotation	Age, weight, height, BMI and walking speed	↑ KOOS in men vs. women (group 2 and 3). ↑ KOOS in women vs. men (group 1). ↑ cadence in women vs. men (group 1 and 3). ↓ knee and hip motion, pelvis rotation in women vs. men (group 1 and 3). ↑ step length in women vs. men (group 2). ↓ pelvis rotation in women vs. men (group 2)	Kiss RM (2011)
66 patients (35 men and 31 women): (1) patients with OA = 30 (16 men and 14 women); (2) controls = 36 (19 men and 17 women)	Cross-sectional study Individuals with knee OA, with at least 50 years and physically active two days a week	-	X-rays Knee	53% (men) and 47% (women)	(1) 66.7 (±7.5) years (men) and 64.3 (±6.4) years (women) (2) 63.5 (±7.5) years (men) and 60.7 (±5.4) years (women)	To identify sex-related differences in muscle activation patterns of patients with OA	BF, MG, LG, hip extension and adduction	Age and BMI	↑ Muscle activity in women vs. men (group 1). ↓ knee joint moments in women vs. men (group 1)	Bigham HJ (2018)
301 patients (134 men and 167 women)	Cross-sectional study Patients with unilateral knee OA and aged 50–85 years	TKA	Not reported Knee	45% (men) and 55% (women)	64.2 (±7.8) years	To investigate sex-affects in the trajectory of functional recovery	Maximal isometric quadriceps and hamstrings contractions, TUG test, SCT and 6 MWT	Age and BMI	↑ Quadriceps and hamstrings strength in women vs. men. ↓ TUG test, SCT and 6 MWT in women vs. men	Gustavson AM (2016)
30 patients (15 men and 15 women): (1) patients with advanced-stage OA = 15 (7 men and 8 women); (2) controls = 15 (8 men and 7 women)	Cohort study Older adults with advanced knee OA and K-L grade 3 or 4	-	X-rays (K-L grading system) and clinical diagnosis of OA Knee	50% (men) and 50% (women)	(1) 71 (±2) years (2) 68 (±1) years	To examine effect of OA on RTD of knee extensors, size and contractility of single muscle fibers	Extensor muscle function and size	Age, height and physical activity	↑ RTD in men vs. women	Callahan DM (2015)
33 patients (16 men and 17 women)	Cohort study Individuals with knee OA and pain	-	ACR clinical criteria and X-rays (K-L grading system) Knee	48% (men) and 52% (women)	62.1 (±7.2) years (men) and 60.4 (±4.3) years (women)	To determine sex differences in quadriceps torque and isotonic power when controlling for muscle volume in patients with OA	Isometric torque, isotonic power and maximal unloaded velocity, voluntary activation, evoked twitch and torque-frequency characteristics	Age, height, weight and BMI	↓ Muscle volume, torque, velocity of contraction, power and peak twitch tension in women vs. men. ↑ half-relaxation time in women vs. men. = voluntary activation and time to peak tension in men and women	Berger MJ (2012)
*Pain*										
2712 patients (1094 men and 1618 women)	Cross-sectional study Individuals aged 50–79 years with knee OA or risk factors (age, female sex, overweight, history of knee symptoms, knee injury and/or surgery)	-	Not reported Knee	40% (men) and 60% (women)	62.0 (±8.3) years (men) and 62.3 (±7.9) years (women)	To determine pain severity in men and women, at equivalent levels of radiographic OA	VAS and WOMAC scores	Age, weight, BMI, race, history of knee symptoms, knee injury and/or surgery, education, comorbidity and analgesic use	↑ Radiographic OA, VAS and WOMAC scores in women vs. men	Glass N (2014)
189 patients (88 men and 101 women)	Cross-sectional study Patients with late-stage hip or knee OA, K-L grade ≥3 and aged ≥35 years	TKA	X-rays (K-L grading system) Hip/knee	47% (men) and 53% (women)	65.66 years (men) and 66.66 years (women)	To investigate sex differences and association between serum levels of CRP and pain in patients with end stage hip/knee OA	CRP and COMP on blood samples, joint and comorbidity counts	Age, BMI, joint/site, comorbidity and serum markers	↑ Joint counts in women vs. men. CRP increase associated to painful joint count increase in women, but not in men	Perruccio AV (2017)
65 patients (37 men and 28 women): (1) pain responder patients; (2) pain non-responder patients	Cross-sectional study Patients with knee OA, older than 18 years, of American Society of Anesthesiologists physical status classes 1 and 2	TKA	Not reported Knee	57% (men) and 43% (women)	-	To analyze inflammatory markers related to acute pain in OA patients	VAS (preoperative and postoperative) and biomarkers on synovial fluids	Age	↑ Probability to report moderate or severe pain in women vs. men. ↑ MMP-10, IL-8, CCL-4, and MCP-2 levels in men vs. women	Solheim N (2017)
179 patients (59 men and 120 women)	Randomized clinical trial Intervention: cognitive behavioral therapy by 8 weekly sessions with a psychologist. Outcome: reduction in sleep disturbances and in OA-related pain Individuals with knee OA, K-L grade >1, pain ratings >2 and insomnia	-	ACR clinical criteria and x-rays (K-L grading system) Knee	33% (men) and 67% (women)	62.78 (±10.11) years (men) and 59.88 (±9.58) years (women)	To examine sex as a moderator of relationships between positive and negative affect, and pain-related outcomes among patients with OA	Positive and negative effects, OA-specific clinical pain, Pain Catastrophizing Scale and QST CS scores	Age, race, marital status, education, sleep disturbance, employment and household income	↑ Positive affect, positive relationship between negative affect and OA-specific pain in men vs. women. ↓ CS, OA-specific clinical pain in men vs. women	Speed TJ (2017)
200 patients (107 men and 93 women)	Cohort study Patients with symptomatic knee OA, vitamin D deficiency, aged 50–79 years, VAS score at least 20 mm, Likert score of 0–2 and serum 25-hydroxyvitamin D levels between 12.5 and 60 nmol/L	-	ACR clinical criteria Knee	53.5% (men) and 46.5% (women)	63.9 (49–79) years (men) and 62.1 (51–78) years (women)	To investigate longitudinal association between endogenous sex hormones, structural changes and pain, in men and women with OA	Cartilage volume and defects, BMLs, effusion-synovitis volume, VAS score, E2, P, T and SHBG serum levels	Age, BMI and sex hormone profiles	↑ T, cartilage and effusion-synovitis volume in men vs. women (at baseline). ↓ SHBG in men vs. women (at baseline). Sex hormones not associated with cartilage volume and defects, BMLs, effusion-synovitis volume and VAS score in men. P positively associated with cartilage volume, E2 negatively associated with BMLs, inverse relationships of sex hormones with effusion-synovitis volume in women	Jin X (2017)
196 patients (81 men and 115 women)	Cohort study Individuals with late-stage knee OA and aged ≥35 years	TKA	Not reported Knee	41% (men) and 59% (women)	64.9 (±8.3) years (men) and 63.4 (±9.0) years (women)	To investigate sex differences in relationship between circulating inflammatory markers and OA pain	IL-6, IL-8, IL-10, IL-1β and TNF-α on blood samples, WOMAC, AOS-Comorbidity Scale, HADS, pain medication use and symptomatic joint count	Age, BMI and comorbidity	↑ Comorbidity and symptomatic joint counts in women vs. men. ↓ depressive symptom and knee pain in women vs. men. Relationships positive for IL-1β and IL-8 in men and negative in women; negative relationship for IL-6 in men and positive for women	Perruccio AV (2019)
42 patients with chronic OA pain (17 men and 25 women)	Cohort study Subjects with knee OA and chronic pain ≥4 of NRS	-	ACR clinical criteria Knee	40% (men) and 60% (women)	54.8 (±68.4) years	To determine the placebo effects in analgesic medication trial on self-reported factors and on performance-based tests for chronic pain due to OA	MPQ-SF, VAS, PASS, CES-D 10, tread-mill distance, sit to stand test, timed stair climb, range of motion and distance from their middle finger to the floor	Age and race	VAS pain intensity decrease and range of motion increase without sex differences. ↑ CES-D 10 depressive symptoms in men vs. women. ↑ treadmill distance in women vs. men	Harden RN (2016)
*Health care needs*										
4478 patients (1708 men and 2770 women)	Cohort study Patients with OA	THA or TKA	Not reported Hip/knee	38% (men) and 62% (women)	64.5 years	To examine sex difference in frequency and types of questions submitted by OA patients	Questions on Anatomy, Condition, Before Surgery, Procedure, After Surgery, Risks and Benefits, and Alternatives to Surgery	Age and surgical procedure	↑ Questions overall number, Condition, Procedure, Risks and Benefits categories in women vs. men	Mora M (2012)
1302 patients (467 men and 835 women)	Cross sectional study Individuals with OA and hip pain	THR	Not reported Hip	36% (men) and 64% (women)	61.8 (±11.6) years (men) and 62.8 (±12.9) years (women)	To examine sex differences along the care pathway	Questionnaire and New Zealand Score	Age, race, status marital, occupation and comorbidity	↓ New Zealand score, pain, comorbidity, THR, probability to have consulted their general practitioner, to have been referred to specialist care, to have consulted an orthopedic surgeon, or to be on a waiting list for THR in men vs. women	Juni P (2010)
244,059 patients (94,695 men and 149,364 women)	Cohort study Patients with knee OA	TKA	Not reported Knee	39% (men) and 61% (women)	64.8 years	To evaluate gender differences in the utilization of OA-related health care resources	Probability to receive narcotic or nonnarcotic analgesic, corticosteroid or hyaluronic acid injection, MRI, physical and occupational therapy	Age, insurance status, region of origin, analgesic use, corticosteroid and hyaluronic acid injections, physical and occupational therapy and walking assistance	↑ Utilization of health care in women vs. men	Bawa HS (2016)
*Arthroplasty*										
963 Japanese patients (368 men and 595 women): (1) patients with <40 = 205 (92 men and 113 women); (2) patients with 40s = 141 (55 men and 86 women); (3) patients with 50s = 192 (62 men and 130 women); (4) patients with 60s = 262 (96 men and 166 women); (5) patients with ≥70 = 163 (63 men and 100 women)	Cross-sectional study Japanese patients with knee OA and K-L grade 2, 3 or 4	-	X-rays (K-L grading system) Knee	38% (men) and 62% (women)	(1) 32.1 years (men) and 33.1 years (women) (2) 45.0 years (men) and 45.0 years (women) (3) 54.4 years (men) and 55.5 years (women) (4) 64.0 years (men) and 64.2 years (women) (5) 75.2 years (men) and 75.0 years (women)	To examine changes and correlations between KSS2011 and KOOS scores by sex, age and severity of OA	KSS2011 and KOOS scores	Age, BMI and K-L grade	↑ OA prevalence in women vs. men. KSS2011 decrease with age no sex-related differences, except for walking and standing score (group 5). KOOS decrease with age and in women over 50, except for sport/recreation	Oishi K (2016)
1254 patients (214 men and 1040 women)	Cohort study Individuals with end-stage knee OA	TKA	Not reported Knee	17% (men) and 83% (women)	67.9 years (men) and 67.3 years (women)	To compare clinical outcomes in men and women	Knee flexion, Oxford Knee Score, KSS and SF-36 scores	Age, race, BMI and mental health	↑ All scores in men vs. women (preoperatively). ↑ Oxford Knee Score and KSS in women vs. men (at 6 months and 2 years). ↑ SF-36 in women vs. men (at 2 years)	Lim JBT (2015)
532 patients (216 men and 316 women)	Cohort study Patients with primary or secondary OA	THA	Not reported Hip	41% (men) and 59% (women)	59 (±16) years (men) and 64 (±14) years (women)	To determine sex differences in patient-perceived functional measures and range of motion	QWB, SF-36, WOMAC, HHS, Merle d’Aubigne’-Postel scores, Hip abduction, adduction, flexion, and internal and external rotation	Age, BMI and comorbidity	↓ All scores in women vs. men (at preoperative). =all scores in men and women (at postoperative). ↑ hip internal rotation in women vs. men	Lavernia CJ (2011)
117 patients (57 men and 60 women): (1) patients with rotator cuff arthropathy = 44; (2) patients with OA and a rotator cuff tear = 73	Cohort study Patients with rotator cuff arthropathy or OA with a rotator cuff tear	RTSA	Not reported Shoulder	49% (men) and 51% (women)	66.9 years (men) and 70.3 years (women)	To determine sex differences in preoperative disability and patient-reported outcomes	Range of motion, VAS, SF-12 MCS, SF-12 PCS, ASES pain and function scores	Age, BMI, smoking history and comorbidity	=Length of stay, demographics, preoperative range of motion, VAS, ASES pain and SF-12 MCS in men and women. ↑ ASES function and SF-12 PCS in men vs. women	Wong SE (2017)
*Other outcomes*										
208 patients (70 men and 138 women)	Cohort study Subjects with unilateral knee OA	TKA	X-rays (K-L grading system) Knee	34% (men) and 66% (women)	61.66 (±9.92) years (men) and 61.92 (±10.03) years (women)	To determine differences on function, physical activity and pain at rest and during movement in men and women with late-stage OA	BPI, KOOS, SF-36, NSR, pressure and heat pain, heat tolerance, GDS, STAI, Pain Catastrophizing Scale, SPS, timed walk, maximal active flexion and extension, physical activity	Age, race, BMI, marital status, education, psychosocial status and analgesic use	↑ BPI in women vs. men. ↓ KOOS, SF-36, pain intensity during the gait speed test and during active knee extension, active knee extension and distance travelled, pain to pressure and heat, and tolerance to heat in women vs. men	Tonelli SM (2011)
289 patients (153 men and 136 women): (1) patients with moderate OA = 83 (51 men and 32 women); (2) patients with severe OA = 143 (76 men and 67 women); (3) healthy controls = 63 (26 men and 37 women)	Cross-sectional study Individuals with unilateral knee moderate or end-stage OA and K-L grade at least 3	-	X-rays (K-L grading system) Knee	53% (men) and 47% (women)	(1) 58.06 (±10.00) years (2) 65.09 (±8.48) years (3) 63.00 (±8.4) years	To quantify gender differences in physical impairments, performance-based measures and patient-reported outcomes with moderate or end-stage OA	Quadriceps strength, TUG, SCT, 6 MWT, KOS-ADLS and PCS of SF-36	Age, BMI and K-L grade	↓ Scores for physical impairment and performance-based measures in women vs. men (all groups). ↓ KOS-ADLS and PCS in women vs. men (group 1)	Logerstedt DS (2014)
504 Korean patients (230 men and 274 women): (1) patients with OA = 188 (36 men and 152 women); (2) patients without OA = 316 (194 men and 122 women)	Cross-sectional study Korean patients with knee OA, K-L grade 2 and aged >50 years	-	X-rays (K-L grading system) Knee	46% (men) and 54% (women)	70.2 years	To investigate the influence of OA on QOL, function, lower extremity physical performance and sex difference	WOMAC and SF-12 scores for pain and function, physical performance test for standing balance, usual walk and chair stands	Age, BMI and K-L grade	↓ Physical role, pain, functioning, mental health, emotional role, vitality, social functioning, PCS and MCS in women vs. men	Kim I (2010)
1348 Japanese patients (452 men and 896 women)	Cohort study Japanese patients with knee OA, K-L grade ≥2 and aged 23–95 years	-	X-rays (K-L grading system) Knee	34% (men) and 66% (women)	64.9 (±11.7) years (men) and 63.3 (±11.8) years (women)	To clarify the associations of physical performance, bone and joint diseases with single and multiple falls	Questionnaire to falls, pain, VFx, grip strength, 6 MWT and chair stand time	Age, height, weight, BMI and region of origin	↑ Radiographic OA, pain, previous falls and VFx in women vs. men (at baseline and 3 years). ↓ grip strength and cognitive impairment in women vs. men (at baseline and 3 years). ↑ 6 MWT (for single falls and multiple falls) in men vs. women. ↓ 6 MWT (for non-falls) in men vs. women. ↑ chair stand time (for non-falls) in women vs. men. ↓ chair stand time (for single falls) in women vs. men	Muraki S (2013)
80 patients (43 men and 37 women): (1) patients with OA = 40 (23 men and 17 women); (2) healthy patients = 40 (20 men and 20 women)	Cross-sectional study Patients with knee OA, pain and aged 25–75 years	-	X-rays Knee	54% (men) and 46% (women)	(1) 64.5 (49–73) years (2) 64.3 (49–73) years	To identify cytokines involved in blood borne, neuroimmune joint-to-CNS signaling in OA patients	Questionnaires (VAS, KOOS pain, SYM, ADL, Sport/Rec, QOL, HADS, PSQI, MFI-20 general, EQ-5D, PPT, PP4 and PP7), biochemical analysis (IL-6, IL-8 and MCP1 levels in CSF, serum and SF) and qRT-PCR (synovial and cartilage mRNA for IL-6, IL-8 and MCP-1)	Age and BMI	↑ VAS, HADS, PSQI, SF IL-8, correlation between CSF MCP-1 and serum, CSF MCP-1 and SF, serum MCP1 and SF, SF MCP-1 and CSF IL-8 in women vs. men (group 1). ↓ PPT, PP4, PP7, KOOS pain, SYM, ADL and Sport/Rec in women vs. men (group 1). Correlations between SF IL-8 and VAS in women, but no in men	Kosek E (2018)

Abbreviations: ↑ = increase; ↓ = decrease; N = number; OA = osteoarthritis; SD = standard deviation; vs = versus; TKA = total knee arthroplasty; MRI = magnetic resonance imaging; BF = biceps femoris; MG = medial gastrocnemius; LG = lateral gastrocnemius; ABD = abductor lever arm; NSA = femoral neck-shaft angle; FNL = femoral neck length; HHC = height of hip center; BWLA = body weight lever arm; FH-FH = femoral head-to-femoral head; HKA = hip-knee-ankle angle; FO = femoral head offset; RTD = rate of torque development; CT = computed tomography; DEXA = dual energy x-ray absorptiometry; AP = anteroposterior; ML = mediolateral; K-L = Kellgren–Lawrence grading scale; WOMAC = Western Ontario McMaster Universities Osteoarthritis Scale; CMC = carpometacarpal; SLS = single-limb-support; DSN = disc space narrowing; OST = osteophytosis; VAS = Pain Visual Analog Scale; TUG = timed Up and Go; SCT = stair-climbing test; 6 MWT = 6-min walk test; TEA = medial and lateral epicondyles; MPQ-SF = McGill Pain Questionnaire Short Form; PASS = Pain Anxiety Symptom Scale; CES-D 10 = Center for Epidemiologic Studies Short Depression Scale; BMLs = bone marrow lesions; E2 = estradiol; P = progesteron; T = testosterone; SHBG = sex hormone binding globulin; THR = total hip replacement; QOL = quality of life; SF-12 = Short Form 12; PCS = Physical Component Summary; MCS = Mental Component Summary; KOOS = Knee Injury and Osteoarthritis Outcome Score; PCA = posterior condylar axis; WSL = Whiteside’s point and femoral center; AP-M = anterior-posterior dimension medial; AP-L = anterior-posterior dimension lateral; CNS = central nervous system; SYM = symptom; ADL = activities of daily living; Sport/Rec = function in sport and recreation; HADS = Hospital Anxiety and Depression Scale; PSQI = Pittsburg Sleep Quality Inventory; MFI-20 = Multidimensional Fatigue Inventory; PPT = Pressure pain thresholds; PP4 = pressure pain corresponding to 4/10; PP7 = pressure pain corresponding to 7/10; IL = Interleukin; MCP = monocyte chemoattractant protein; CSF = cerebrospinal fluid; SF = synovial fluid; qRT-PCR = Quantitative real-time polymerase chain reaction; THA = total hip arthroplasty; QWB = Quality of Well-being; SF-36 = Short Form 36; SBC = subchondral bone cyst; CV/TV = cyst volume/total volume; BV/TV = bone volume fraction; Tb.Th = trabecular thickness; Tb.Sp = trabecular separation; Tb.N = trabecular number; SMI = structure model index; DA = degree of anisotropy; Conn.D = connectivity density; BMD = bone mineral density; STB = subchondral trabecular bone; DTB = deeper trabecular bone; O.Th = thickness of osteoid; OV/BV = percent osteoid volume; OS/BS = percent osteoid surface; OS/BV = specific osteoid surface; ES/BS = percent eroded surface; ES/BV = specific eroded surface; ES/TV = eroded surface in bone tissue volume; KSS = Knee Society Score; KOS-ADSL = Knee Outcome Surveye Activities of Daily Living Scale; 3D = three dimensional; JSN = joint space narrowing; FTA = femorotibial angle; VFx = vertebral fracture; AA = African-American; WH = White; AD = acetabular depth; AW = acetabular width; AI = acetabular index; LCEA = lateral center edge angle; MTHI = modified triangular index height; PFA = proximal femoral angle; FSA = femoral shaft angle; fJSW = fixed joint space width; RA = rheumatoid arthritis; KSS2011 = 2011 Revised Knee Society Score; KAM = knee adduction moment; GRF = ground reaction force; CRP = C-reactive protein; COMP = cartilage oligomeric matrixprotein; TNF-α = tumor necrosis factor α; MMP = metalloproteinase; QST = Quantitative sensory testing; CS = central sensitization; BPI = Brief Pain Inventory; RTSA = Reverse total shoulder arthroplasty; ASES = American Shoulder and Elbow Surgeons; LAP = lateral anteroposterior; MAP = medial anteroposterior; NRS = numerical rating scale; SI = sacroiliac; P1/3 = proximal one-third plane; DL = distal plane under lesser trochanter; GDS = Geriatric Depression Scale; STAI = State Trait Anxiety Inventory; SPS = Social Provisions Scale; HHS = Harris hip score; BMI = body mass index; ACR = American College of Rheumatology.
